# Medical Association of Georgia

**Published:** 1891-05

**Authors:** 


### Society Notes.

### MEDICAL ASSOCIATION OF GEORGIA.

FIRST DAY—*M rning Session.*

Forty-second annual meeting, held in Augusta, April 15, 16, and 17, 1891.

Society met at Masonic Hall, and was called to order by the President, Dr. A. W. Griggs, of West Point, at 10 o'clock a. m.

Prayer by Rev. George W. Yarbrough, of Augusta.

Address of welcome in behalf of the medical profession of Georgia by Dr. Thomas D. Coleman.

Response in behalf of Medical Association by Willis F. Westmoreland, of Atlanta.

The President then delivered his annual address.

Dr. Eugene Foster, as chairman of the committee on programme, submitted his report, which was accepted and carried out the following order:

Filling vacancies in Board of Censors.

Application for membership.

Report of Secretary.

Report of Treasure .

Appointment of committee to audit books of Treasurer.

Adjournment.

AFTERNOON SESSION.

1. Meeting called to order promptly at 3:30 p. m.
2. Report of the Board of Censors.
3. Reading paper, by Dr. T. R. Wright, of Augusta, on "Report of Surgical Cases." Leaders of discussion, Drs. Thomas D. Coleman and G. C. Dugas.
4. 5 o'clock p. m.—Reading paper, by Dr. W. F. Westmoreland, of Atlanta, on "Spasmodic Stricture of Urethra." Leaders of discussion, Drs. R. O. Ingram, H. F. Campbell and J. F. Lancaster.
5. Adjourned.

The papers of both Dr. Wright and Dr. Westmoreland proved very interesting, and caused much discussion.

After the adjournment many of the delegates were shown over the city by their local colleagues.

---

SECOND DAY—*Morning Session.*

Thursday, April 16. The second day of the meeting was quite interesting, and the Association was called to order promptly at 9 a. m. by the president.

The minutes of the previous day were read.

Applications for membership presented; the report of the Censors received, then Dr. T. M. Holmes read his paper on "A Case of Sciatica Terminating in Death." This paper called forth quite a lengthy discussion.

Dr. Eugene Foster followed in a paper entitled "Modern Surgery in Relation to Wound Treatment." Quite a spirited debate followed this paper between the believers in antiseptics and the non-believers.

Dr. W. L. Holliday presented a voluntary paper on "A Case of Enormously Distended Bladder in Female Infant Displaced through the Vagina, and Treated by Extra-Vaginal Tapping."

This was followed by the orator's address, Dr. J. Lindsay Johnson, of Cartersville, being the honored man.

During the afternoon session the standing committees reported; the committee to nominate officers was appointed; applications for membership received, and the report of the Board of Censors accepted.

Dr. Thomas D. Coleman's paper on "Phthisis Pulmonalis," was then read, and was ably discussed pro and con.

Dr. J. M. Hull's paper on "Absorption of Cataract," was well received.

Dr. E. H. Richardson, of Atlanta, Ga., presented a paper—"When shall we Interfere in Cases of Threatened Puerperal Convulsions?" This paper brought out a great deal of discussion.

The Association then adjourned.

This evening one of the most delightful banquets ever given the Association was enjoyed by the members present. How well it was enjoyed, I will not attempt to say—better "ask of the winds;" they know more than I.

---

THIRD DAY—*Morning Session.*

Friday, April 17. The last day of the meeting opened with a very good attendance, and a number of very interesting papers were read, but Augusta's hospitality of the previous night had had its effect on many of the leading lights.

The paper of Dr. DeSaussure Ford, on "The Extirpation of two inches of the Popliteal Nerve for the Relief of Neuralgia," called forth a very warm discussion. This was followed by a paper by Dr. R. J. Nunn, on "A Treatment for Melionhagia," which was ably discussed by the members present.

Dr. C. C. Fowler gave an interesting report of a case of "Battey's Operation."

At the afternoon session Dr. T. M. Holmes read an excellent paper entitled "An Obstinate Case of Dysentery, and Compress as a Dernier Resort."

Dr. K. P. Moore, the efficient secretary for the last three years, now offered his resignation, which was accepted with regret by the Association.

The most interesting feature of the day was now taken up, the election of officers for the ensuing year, which resulted as follows ;

President—Dr. G. W. Mulligan, of Washington.

First Vice-President—Dr. J. M. Hull, of Augusta.

Second Vice-President—Dr. Mark O'Daniel, of Milledgeville.

Secretary—Dr. Dan. H. Howell, of Atlanta.

Treasurer—Dr. E. C. Goodrich, of Augusta.

Censors—Drs. Eugene Foster and K. P. Moore.

This meeting is one of the best the Association has ever had, and many new members were added to the already large list of membership.

The next meeting will be held in Columbus, and many have already stated that they will be there.

After adjournment, many tarried, as if loth to leave the Fountain City.

